# Advances in non-invasive brain stimulation: enhancing sports performance function and insights into exercise science

**DOI:** 10.3389/fnhum.2024.1477111

**Published:** 2024-11-29

**Authors:** Shuo Qi, Jinglun Yu, Li Li, Chen Dong, Zhe Ji, Lei Cao, Zhen Wei, Zhiqiang Liang

**Affiliations:** ^1^School of Sport and Health, Shandong Sport University, Jinan, China; ^2^College of Sports and Health Sciences, Xi’an Physical Education University, Xi’an, China; ^3^Physical Education and Arts College, Shandong Sport University, Jinan, China; ^4^College of Sports Management, Shandong Sport University, Jinan, China; ^5^College of Physical Education, Anhui Normal University, Wuhu, China; ^6^National Football Academy, Shandong Sport University, Jinan, China; ^7^The Second Clinical Medical School, Xuzhou Medical University, Xuzhou, China; ^8^Faculty of Sports Science, Ningbo University, Ningbo, China

**Keywords:** non-invasive brain stimulation, motor performance, exercise science, athletic training optimization, neuroplasticity and exercise, temporal interference stimulation

## Abstract

The cerebral cortex, as the pinnacle of human complexity, poses formidable challenges to contemporary neuroscience. Recent advancements in non-invasive brain stimulation have been pivotal in enhancing human locomotor functions, a burgeoning area of interest in exercise science. Techniques such as transcranial direct current stimulation, transcranial alternating current stimulation, transcranial random noise stimulation, and transcranial magnetic stimulation are widely recognized for their neuromodulator capabilities. Despite their broad applications, these methods are not without limitations, notably in spatial and temporal resolution and their inability to target deep brain structures effectively. The advent of innovative non-invasive brain stimulation modalities, including transcranial focused ultrasound stimulation and temporal interference stimulation technology, heralds a new era in neuromodulation. These approaches offer superior spatial and temporal precision, promising to elevate athletic performance, accelerate sport science research, and enhance recovery from sports-related injuries and neurological conditions. This comprehensive review delves into the principles, applications, and future prospects of non-invasive brain stimulation in the realm of exercise science. By elucidating the mechanisms of action and potential benefits, this study aims to arm researchers with the tools necessary to modulate targeted brain regions, thereby deepening our understanding of the intricate interplay between brain function and human behavior.

## 1 Introduction

Neuroscience has emerged as a pivotal area of research within the life sciences, with brain science representing a critical subfield. Brain stimulation techniques serve as essential tools for neuromodulation, significantly contributing to our understanding of brain function, enhancement, protection, and simulation. These methods hold considerable promise for the treatment of neurological disorders and the enhancement of cognitive and physical performance (Qi et al., 2024). Brain stimulation techniques can be categorized into two primary types: invasive and non-invasive. Traditional non-invasive brain stimulation (NIBS) methods include transcranial direct current stimulation (tDCS), transcranial alternating current stimulation (tACS), transcranial magnetic stimulation (TMS), and transcranial random noise stimulation (tRNS) (Camacho-Conde et al., 2022). Each of these modalities exhibits distinct output characteristics and mechanisms of action.

Nowdays, invasive techniques like deep brain stimulation (DBS) are used to treat motor deficits in neurological conditions like essential tremor and dystonia, but they come with surgical risks such as intracranial hemorrhage, infection, and electrode displacement, limiting their use to patients and hindering research in healthy populations (Bhattacharya et al., 2021). With advancements in brain stimulation technologies, novel non-invasive techniques have emerged, such as transcranial focused ultrasound stimulation (tFUS) and temporal interference stimulation (TIS), which allow for targeted stimulation of deeper brain regions (Cao and Grover, 2020; Dmochowski and Bikson, 2017; Mirzakhalili et al., 2020; Zhou et al., 2019). These innovative neuromodulation strategies promise precise, non-invasive brain function modulation with minimal side effects, overcoming traditional stimulation limitations by targeting deep tissues without disrupting cortical activity.

Athletic performance is crucial not only for athletes but also influences the combat readiness of military personnel, the developmental trajectories of children and adolescents, and the health management of the elderly, thereby reflecting societal productivity and national competitiveness. Recent research in exercise science has found that NIBS enhances neuromuscular coordination during physical activity by strengthening the connections between the brain, nerves, and muscles, demonstrating potential for improving various aspects of athletic performance, including balance, endurance, fatigue resistance, muscle strength, and motor learning (Grosprêtre et al., 2021; Maudrich et al., 2022). Among these NIBS, the tDCS has garnered significant attention for its ability to enhance muscle strength and motor perception, delay fatigue, and facilitate motor skill acquisition in healthy individuals and athletes.

This review aims to synthesize current knowledge on prevalent non-invasive neuromodulation techniques, alongside emerging non-invasive deep brain stimulation modalities within the sports science. By highlighting innovative methodologies and perspectives, this study seeks to contribute to the advancement of sports science research and the enhancement of human athletic performance.

## 2 Traditional non-invasive brain stimulation modalities

### 2.1 Transcranial direct current stimulation and its impact on athletic performance

#### 2.1.1 tDCS and its mechanism

**Figure 1:**
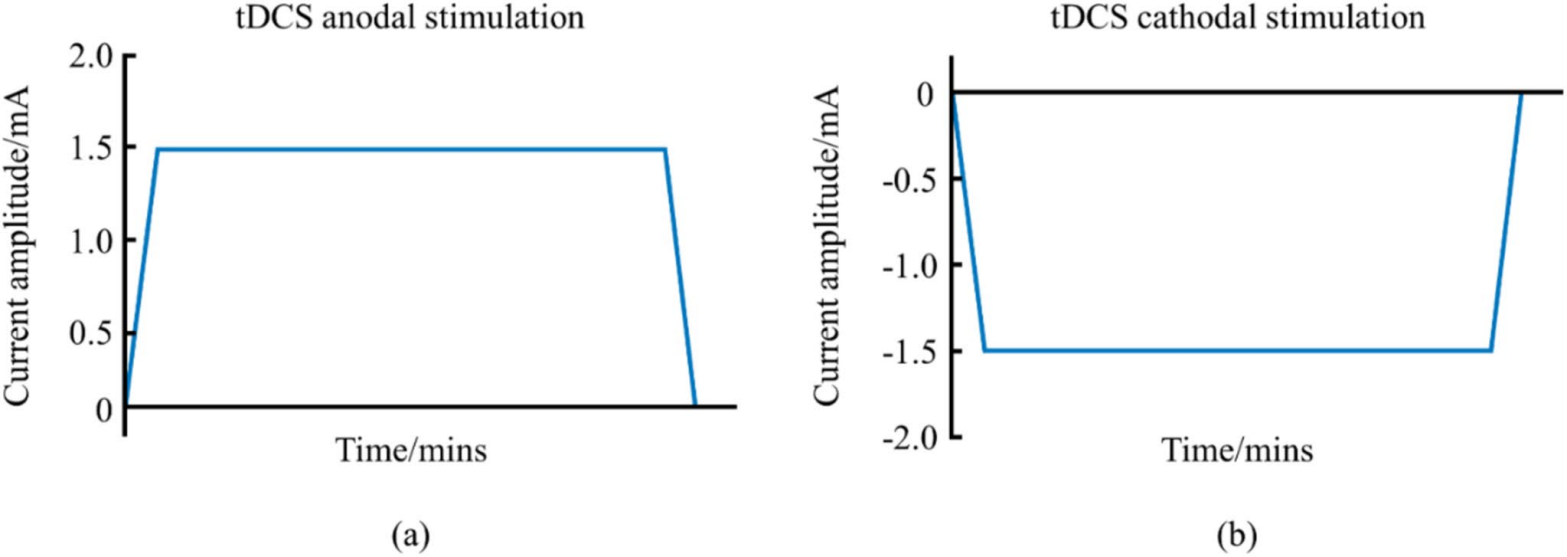
Schematic diagram of the waveform of tDCS. (a) Represents the anodal tDCS waveform schematic. (b) Represents the cathodal tDCS waveform schematic.

tDCS, a prominent NIBS, employs a continuous low-intensity direct current (1–2 mA) to modulate cortical neuronal activity, thereby directly influencing athletic performance (Reardon, 2016). This system, comprising a battery-operated stimulator and two electrodes—an anode and a cathode—positioned on the scalp, delivers a steady current that can enhance muscle strength, motor skills, and overall athletic performance (Jamil et al., 2017). tDCS can be categorized into anodal and cathodal types, with waveform variations shown in Figure 1. This system, comprising a battery-operated stimulator and two electrodes—an anode and a cathode—positioned on the scalp, delivers a steady current that can enhance muscle strength, motor skills, and overall athletic performance.

**Figure 2:**
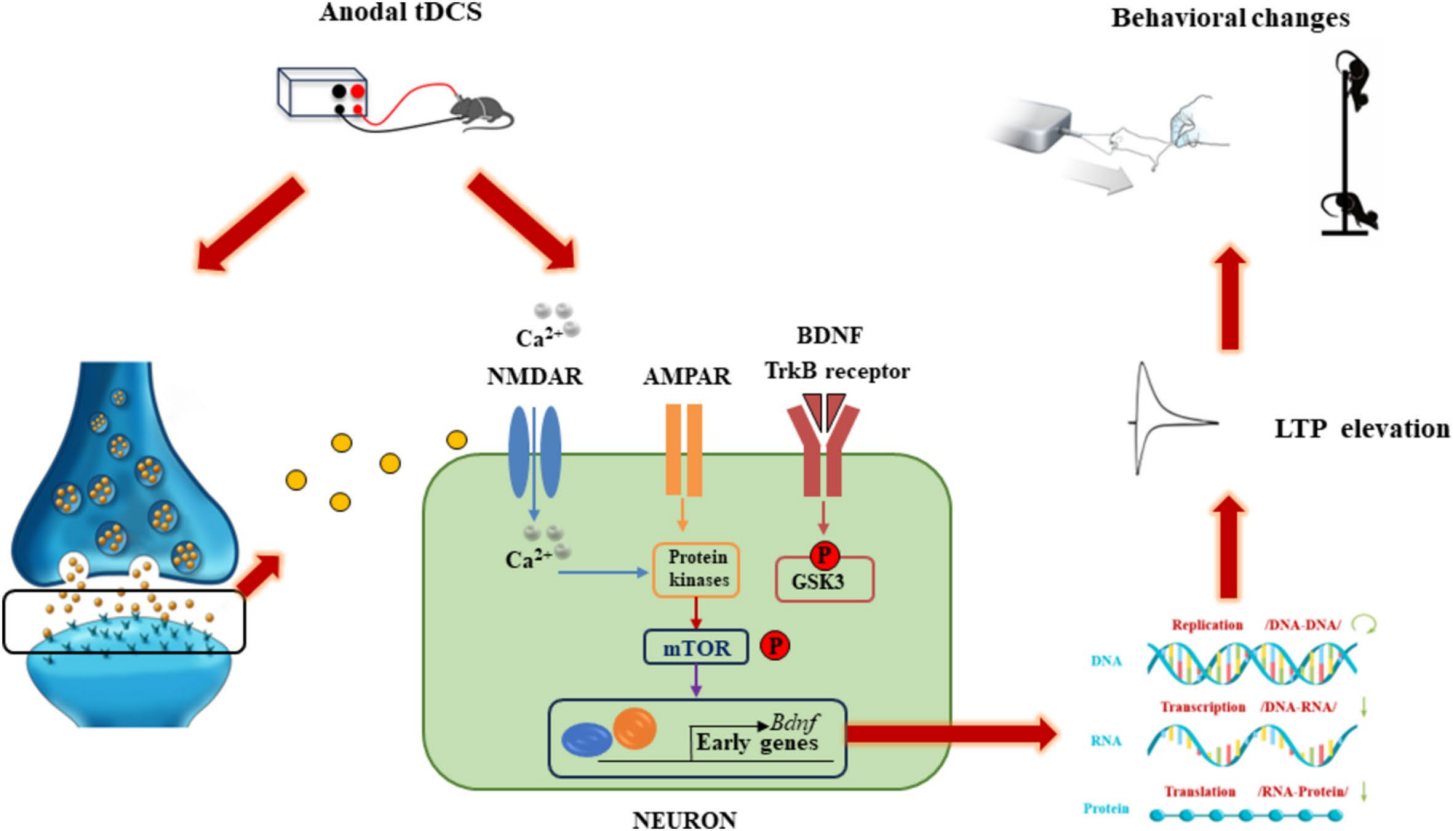
Schematic diagram of the molecular mechanism of tDCS action.

The application of direct current in tDCS travels through the scalp and penetrates the outer cortical layers, directly influencing the membrane potential of neurons within targeted cortical regions, which is crucial for enhancing athletic performance (Bhattacharya et al., 2021). The current flow from the anode to the cathode induces alterations in the electrical activity of neurons, thereby modifying synaptic efficiency and leading to improved motor learning and enhanced athletic performance. The synapse plays a crucial role in integrating neural signals and facilitating cellular connections, and is the basic unit of neural circuit activity. It plays a key regulatory role in neural communication. Synapses are not static structures, but are constantly being altered by various stimuli. External stimuli activate neurons throughout the brain, leading to structural and functional changes in synapses called synaptic plasticity (Citri and Malenka, 2008; Martin et al., 2000; Takeuchi et al., 2014). Synaptic plasticity can lead to structural or functional reorganization of neurons (Appelbaum et al., 2023). The best-known form of functional plasticity is the long-term potentiation (LTP)-like effect, characterized by sustained synaptic reinforcement, which is a key mechanism for learning and memory (Choi and Kaang, 2022). Anodal tDCS can lead to depolarization of neuronal cell membranes, which promotes neurotransmitter release from the presynaptic membrane. As shown in Figure 2, this process engaged N-methyl-D-aspartic acid receptors (NMDAR) and alpha-amino-3-hydroxy-5-methyl-4-isoxazolepropionic acid receptors (AMPAR) on the postsynaptic membrane, resulting in an upregulation of intracellular calcium ions and the subsequent activation of protein kinases. This cascade can enhance the production of brain-derived neurotrophic factor (BDNF) via modulation of the mTOR signaling pathway. Over time, these processes can lead to increased gene transcription and the synthesis of proteins that facilitate LTP-like effect and improve behavioral outcomes (Bandeira et al., 2021; Cavaleiro et al., 2020). The synaptic plasticity induced by tDCS encompasses multiple aspects of neurobiology and neurophysiology, including gene transcription, protein expression, neurotrophic factor regulation, neural signaling and synaptic remodeling. Given the important role of LTP and long-term depression (LTD)-like effects in synaptic plasticity, the researchers suggest that tDCS may induce long-term changes in brain excitability and activity through LTP and LTD-like effects. This mechanism contributes to enhanced synaptic plasticity and ultimately improved motor performance.

#### 2.1.2 tDCS application and its impact on athletic performance

In recent years, leading journals like ***Nature*** have published several articles focusing on tDCS and its direct applications in enhancing athletic performance, demonstrating the practical relevance of this basic research to sports science. For instance, in 2016, Reardon reported on a collaboration between the US Ski Association and Halo Neuroscience, which developed an electroencephalographic stimulation device designed to enhance explosive power by delivering mild electrical currents to the brain via stimulation electrodes integrated into headphones. This approach aims to stimulate brain regions responsible for movement, flexibility, and other motor functions (Reardon, 2016). The headset was fundamentally based on tDCS technology. In subsequent research, Hornyak characterized tDCS as a novel “neural priming technique” that enhanced motor performance by improving the nerve–muscle connection, facilitating neuromuscular recruitment, and optimizing coordination among muscle groups in athletes (Hornyak, 2017). As brain science research advances, tDCS has gained traction in both clinical and translational studies, finding applications in military and competitive sports settings (Bestmann and Walsh, 2017). For example, the United States Snowboard Association incorporated tDCS training for Winter Olympic ski jumpers in 2016 to boost the athletes’ explosiveness and coordination (Reardon, 2016). Additionally, professional sports organizations, including the National Football League and the National Basketball Association, as well as Olympic cyclists and triathletes, have utilized Halo Sport headphones based on tDCS principles to enhance their performance (Garner et al., 2021).

Research within sports science has demonstrated that muscle strength can be significantly enhanced following anodal tDCS intervention by increasing the excitability of corticospinal tract conduction (Angius et al., 2016; Lattari et al., 2020). For example, 10 min of anodal tDCS has been shown to improve ankle plantar flexor strength (Tanaka et al., 2009), while a 20-min intervention can enhance the maximal voluntary contraction of wrist extensors, biceps brachii, and knee extensors (Flood et al., 2017; Fridriksson et al., 2018; Hazime et al., 2017). Muscle strength is a critical component for athletes in executing technical movements and achieving rapid performance during competitions. Moreover, anodal tDCS has been found to enhance the muscle strength of adolescent soccer players, aiding their execution of various sports skills (Vargas et al., 2018). For the general population, anodal tDCS also effectively improves muscle strength and physical fitness in healthy individuals, manifesting in increased muscle strength, load capacity, and the number of repetitions at maximal strength (Lattari et al., 2016). Additionally, Xiao et al. (2020) observed that high-precision tDCS stimulation of the sensorimotor region in healthy adults for 20 min led to significant improvements in static balance, a fundamental skill for athletes, particularly in non-rhythmic sports where balance directly influences performance (Xiao et al., 2020). Future research is warranted to evaluate the effects of tDCS on dynamic balance in elite athletes. Motor skills, defined as the ability to learn and execute specific movements, rely on synaptic plasticity and functional connectivity across various cortical regions. tDCS has been shown to enhance motor skill acquisition and consolidation in healthy individuals (Besson et al., 2020; Kaminski et al., 2021). For example, Zhu et al. (2015) found that cathodal stimulation of the left dorsolateral prefrontal cortex inhibited verbal working memory activity, reducing episodic verbal analyses during motor control, thereby improving the golf putting performance of healthy college students (Zhu et al., 2015). tDCS can alter the excitability by regulating the resting membrane potential of neurons, and studies have shown that when the primary motor cortex (M1) region of the brain is used as the stimulation region, the anodal tDCS placed above the M1 region modulates the resting membrane potential of neurons and approaches depolarization and increase the excitability of the cerebral cortex, influencing spinal cord neural pathways, enhancing motor unit recruitment, and ultimately enhancing motor performance (Angius et al., 2016; Lattari et al., 2020).

#### 2.1.3 The adverse effects of tDCS

A comprehensive review of the adverse effects of tDCS on motor and non-motor cortical areas in both healthy participants and psychiatric patients indicated that serious adverse events were rare across approximately 18,000 tDCS interventions. Mild side effects reported included fatigue, headache, and minor sensations of itching or burning (Antal et al., 2017). While tDCS is widely recognized as a safe and non-invasive technique for enhancing athletic performance, its broad stimulation characteristics limit its ability to specifically target deep brain tissues. Despite the growing body of research on tDCS, the precise neurophysiological mechanisms underlying its effects remain incompletely understood, warranting further investigation to elucidate these processes.

### 2.2 Transcranial alternating current stimulation

#### 2.2.1 tACS and its mechanism

**Figure 3:**
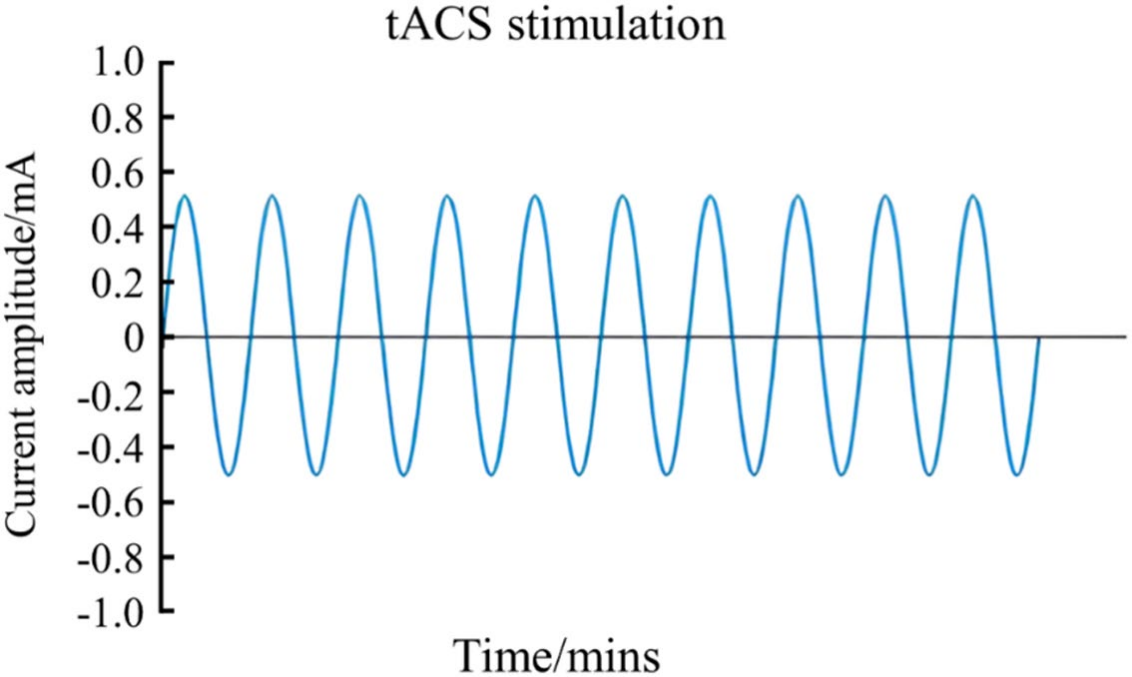
Schematic diagram of the working principle of tACS.

tACS is a NIBS technique that delivers oscillating currents to the brain, thereby modulating neuronal activity (Antal et al., 2008; Antal and Paulus, 2013; Guerra et al., 2018). This method operates by applying a sinusoidal current through two electrodes placed on the scalp, which alternates in polarity according to a sinusoidal wave pattern (Antal and Herrmann, 2016). The alternating current (AC) penetrates the skull and influences cortical neurons (Figure 3). The sinusoidal waveform of tACS results in voltage fluctuations that vary gradually from positive to negative in each half cycle (Elyamany et al., 2021). The parameters of tACS, including electrode placement, current characteristics such as frequency and amplitude, are critical for its effectiveness (Huang et al., 2018; Woods et al., 2016). Typically, the stimulation frequency is aligned with the corresponding electroencephalography (EEG) frequency to modulate related brain processes (Elyamany et al., 2021).

tACS has been associated with distinct frequency bands commonly recognized in neurophysiological research. These include the theta band (4–7 Hz), alpha band (8–13 Hz), beta band (14–30 Hz), and gamma band (31–100 Hz). The 0–4 Hz frequency band has traditionally been linked to deep sleep and memory consolidation but has recently been associated with attention and rhythm-dependent cognitive processes (Morillon et al., 2019). The 4–7 Hz oscillations primarily contribute to working memory and episodic memory (Herweg et al., 2020), while the 8–13 Hz range is involved in executive functions, visual attention, and memory processes (Kim et al., 2017; Mierau et al., 2017). The beta band (14–30 Hz) is crucial for motor and cognitive functions related to working memory and executive control, whereas gamma band oscillations (31–100 Hz) are primarily responsible for processing sensory information and episodic memory (Fries, 2015; Pina et al., 2018), as well as auditory perception (Baltus and Herrmann, 2016).

#### 2.2.2 tACS application in motor learning and athletic performance

Recent studies have shown that tACS can enhance sequence learning and motor skill acquisition, which are fundamental abilities that translate into broader athletic performance. Investigations by Pollok et al. (2015) explored the effects of alpha-band and beta-band tACS applied to the left primary motor cortex (M1) during motor sequence task learning (Pollok et al., 2015). Their findings indicated significant improvements in serial reaction time task (SRTT) performance at both 10 Hz and 20 Hz stimulation frequencies, suggesting that tACS enhances sequence learning and motor skill acquisition. Similarly, Sugata et al. (2018) and Giustiniani et al. (2019) reported that tACS stimulation of M1 at frequencies of 40 Hz and 70 Hz facilitated improvements in motor learning (Giustiniani et al., 2019; Sugata et al., 2018). Furthermore, Miyaguchi et al. (2020) found that simultaneous application of 70 Hz tACS to both the M1 and the cerebellum promoted retention of fine motor skills in a visuomotor control task (Miyaguchi et al., 2020). Neuroimaging evidence suggests that gamma-band tACS targeting M1 enhances motor learning by decreasing inhibition of γ-aminobutyric acid type A (GABA-A) receptors in the local resting state (Nowak et al., 2017).

When tACS was applied to the scalp, a portion of the current penetrates the brain, leading to oscillatory changes in the membrane potential of cortical neurons, resulting in depolarization or hyperpolarization (Vöröslakos et al., 2018). Although this oscillatory change in membrane potential is sufficient to induce action potentials, it does not significantly alter the rate of neuronal firing; rather, it modulates the timing of action potentials in a frequency- and location-specific manner (Krause et al., 2019; Vosskuhl et al., 2018). The cellular signaling mechanisms underlying tACS are associated with the induction of LTP- and LTD-like effects. tACS promotes an increase in intracellular calcium ion concentrations, activating calcium-dependent enzymes. This presynaptic mechanism facilitates glutamate release and activates AMPAR and NMDAR, modulating BDNF release and interaction with TrkB receptors, which collectively lead to intracellular events that support *de novo* protein synthesis and the establishment of LTP and LTD.

The enhancement of motor performance by tACS can be achieved through a variety of mechanisms, including the following: modulation of electrical brain activity: tACS can enhance neural activity in motor-related brain regions by modulating brain waves at specific frequencies (Antal and Paulus, 2013; Vogeti et al., 2022). This modulation helps to improve motor control and coordination. Neuroplasticity: tACS promotes neuroplasticity, which helps the brain to reorganize and optimize neural connections when learning new skills, thus enhancing motor learning and memory (Elyamany et al., 2021). However, given the variability in tACS stimulation protocols, further large-scale studies are necessary to validate these findings. Despite substantial research advancements in the field of neuromodulation, the effects of tACS require additional investigation at multiple levels, from molecular mechanisms to animal neurophysiology and clinical applications. Future research endeavors that integrate neurophysiological techniques and brain imaging with traditional electrical nerve stimulation methods are crucial for elucidating the neurophysiological mechanisms of tACS and expanding its potential clinical applications.

### 2.3 Transcranial random noise stimulation

**Figure 4:**
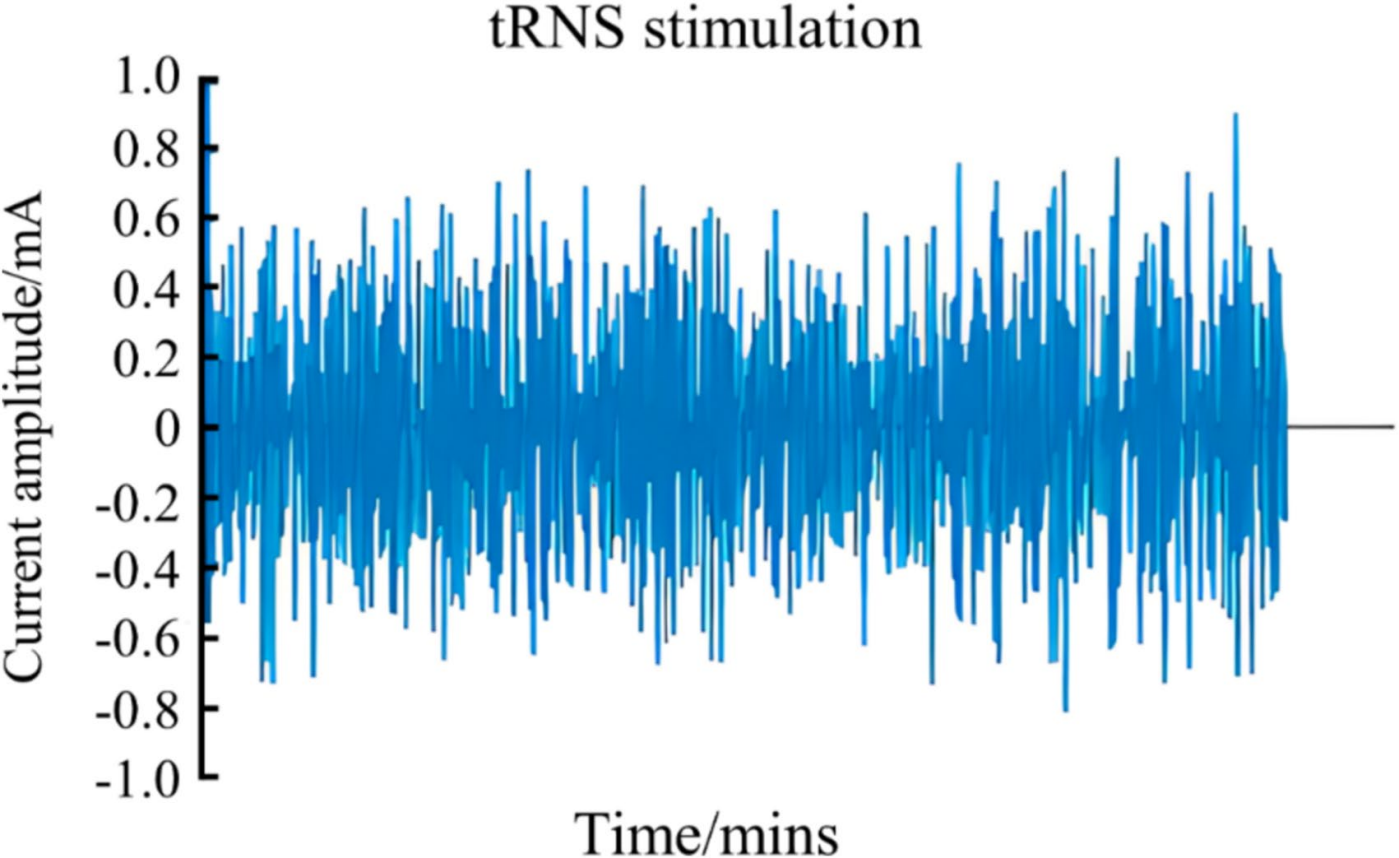
Schematic diagram of tRNS waveform.

tRNS is a non-invasive brain stimulation technique that generates low-intensity, randomly varying frequency and amplitude currents, resembling bell-shaped or normally distributed white noise. These currents can be classified into low-frequency (0.1–100 Hz), high-frequency (101–640 Hz), and full-frequency (0.1–640 Hz) categories (Figure 4) (Antal and Herrmann, 2016; Reed and Cohen Kadosh, 2018). The application of high-frequency tRNS to the motor cortex has been shown to significantly increase motor cortical excitability, with effects persisting for up to 60 min post-stimulation (Kortuem et al., 2019; Moliadze et al., 2014). This enhancement in motor cortical excitability, as demonstrated by improved task accuracy and reduced reaction times in GO/NO-GO tasks, can have direct implications for sports performance, where rapid and accurate motor responses are crucial for success (Andreas et al., 2019). These findings suggest that tRNS can enhance fundamental motor learning abilities, which are essential for high-level athletic performance (Jooss et al., 2019). According to previous study, tRNS may alter excitability by modulating the resting membrane potential of neurons, affecting spinal cord neural pathways, enhancing corticospinal excitability, enhancing motor unit recruitment, and ultimately improving motor function (Hoshi et al., 2021).

Additionally, high-frequency tRNS applied to the left motor cortex for 10 min significantly reduced error rates during a visual eye-muscle tracking task, further supporting the notion that tRNS can facilitate motor learning (Abe et al., 2019). However, although existing literature on the impact of tRNS on motor learning remains relatively limited, current hypotheses suggest that tRNS may modulate cortical excitability by influencing voltage-gated sodium channels (Chaieb et al., 2015; Potok et al., 2022; Remedios et al., 2019). Hence, future systematic investigations across diverse populations and various learning paradigms are necessary to establish the conditions under which tRNS can effectively enhance learning outcomes and, by extension, athletic performance.

### 2.4 Transcranial magnetic stimulation

TMS is a non-invasive neuromodulation technique grounded in the principles of electromagnetic induction (Bhattacharya et al., 2021). It has been extensively utilized to investigate intracortical, cortico-cortical, and cortico-subcortical interactions within the brain (Moscatelli et al., 2021). The foundational effects of TMS on the human motor cortex were first documented by Barker et al. (1985).

TMS encompasses three primary stimulation modalities: single-pulse TMS, double-pulse TMS, and repetitive TMS (rTMS). rTMS, in particular, has broadened the clinical applications of magnetic stimulation and is among the most widely employed techniques in current practice (Bhattacharya et al., 2021). High frequency rTMS acting on the dorsolateral prefrontal cortex (DLPFC) of volleyball players can improve body coordination and enhance athletic performance (Moscatelli et al., 2023). Single-pulse TMS evaluates motor cortical excitability through parameters such as the amplitude of motor evoked potentials (MEPs) and active motor thresholds. In contrast, double-pulse TMS is utilized to assess inhibitory and facilitatory intracortical circuits, quantifying phenomena such as short-interval cortical inhibition and intracortical facilitation (Agrawal et al., 2021).

**Figure 5:**
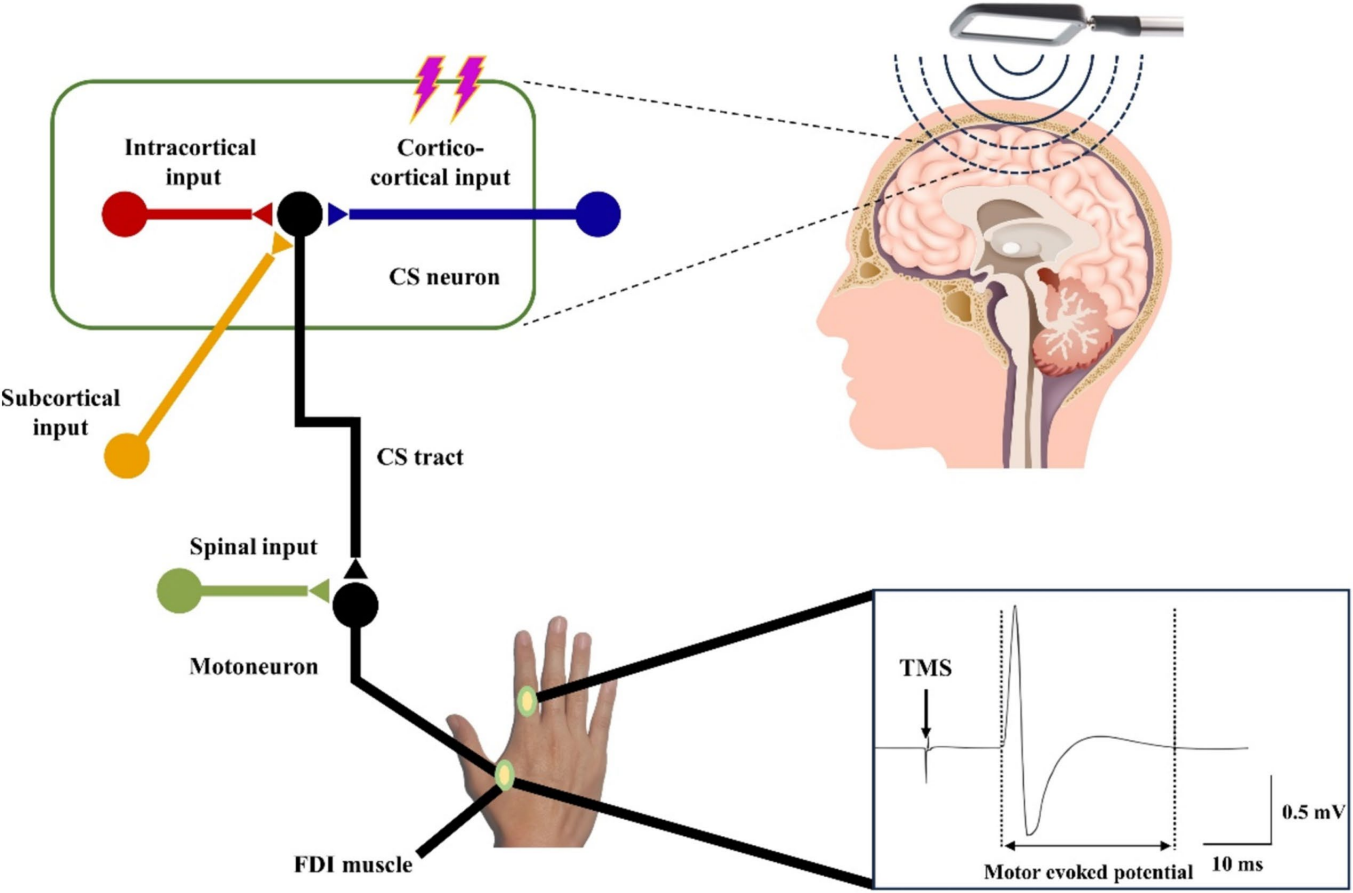
Schematic diagram of the working principle of TMS.

The TMS apparatus consists of a series of capacitors connected to a coil of wire, characterized by its inductance and resistance. When a rapidly changing current traverses the coil placed on the scalp, it generates a varying magnetic field that penetrates the skull, producing eddy currents. These currents subsequently induce action potentials in specific brain regions (Bhattacharya et al., 2021). As illustrated in Figure 5, the magnetic field produced by the coil positioned over the hand representation in the M1 activates cortical circuits, which in turn stimulate corticospinal neurons and alpha motor neurons in the spinal cord, innervating muscles such as the first dorsal interosseous. This activation is recorded as MEPs through surface electromyographic (EMG) signals (Bhattacharya et al., 2021). TMS can increase the excitability of the spinal cord, thereby recruiting more spinal motor neurons, causing an increase in the synchrony of spinal motor neuron firing, enhancing motor unit recruitment, and ultimately improving motor function (Moscatelli et al., 2021).

In recent years, the application of TMS within the field of exercise science has garnered increasing interest. Researchers have employed TMS to explore central fatigue, sensorimotor integration, motor coordination, and neuronal plasticity post-exercise (Moscatelli et al., 2021). Evidence suggests that TMS applied to the cerebral cortex can enhance grip strength, likely related to the extent of motor unit recruitment induced by the stimulation (Cros et al., 2007). However, the range of induced currents and the specific types of neurons targeted by TMS remain ambiguous, as do the excitatory, inhibitory, or state-dependent effects of TMS on these neurons (Moscatelli et al., 2021). Another limitation of TMS is its primary focus on cortical regions, akin to tDCS and tACS, which makes it challenging to stimulate deeper brain structures (Deng et al., 2013; Moscatelli et al., 2021).

Despite these limitations, TMS is recognized as a safe non-invasive brain stimulation therapy that is increasingly integrated into clinical practice, demonstrating efficacy in neurorehabilitation, psychosomatic treatments, and brain function assessments. A deeper understanding of TMS’s features and mechanisms is anticipated to broaden its application spectrum. Moreover, the integration of TMS with electrophysiological recordings and functional magnetic resonance imaging (fMRI) has the potential to facilitate real-time monitoring of neuronal activity and provide precise guidance for stimulation localization, thereby enhancing the overall efficacy of the intervention.

## 3 Non-invasive brain stimulation modalities that can be focused deep into the brain

### 3.1 Transcranial focused ultrasound stimulation

**Figure 6:**
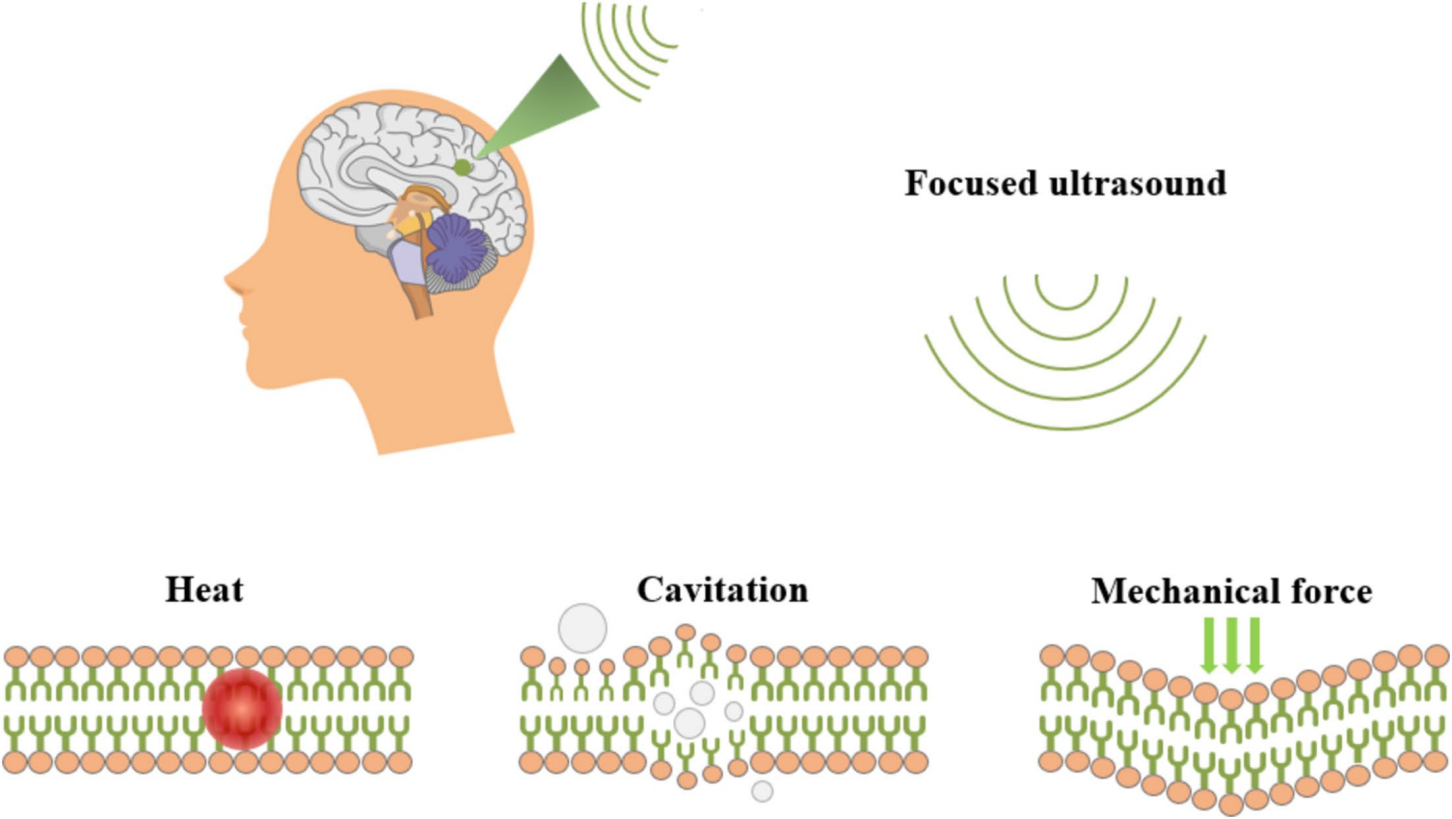
Schematic diagram of tFUS ultrasonic neuromodulation.

tFUS is a non-invasive neuromodulation technique that leverages ultrasonic mechanical effects to target and modulate deep brain structures with high spatial and temporal resolution, as well as significant penetration depth (Constans et al., 2020; Folloni et al., 2019; Li et al., 2019; Tyler et al., 2018; Zhou et al., 2019). As illustrated in Figure 6, tFUS penetrated biological tissues in deep brain regions, delivering mechanical forces that create focal thermal and mechano-biological effects (Blackmore et al., 2019; Kubanek, 2018). As a conducted wave, ultrasound can alter neuronal and muscle activity by stimulating nerves and muscle fibers. Fry were the first to report that ultrasound significantly influences neuronal activity in the brain, highlighting its potential for treating movement disorders and chronic pain through high-intensity focused ultrasound stimulation (Fry, 1958; Liu et al., 2021).

tFUS holds considerable promise for both neuroscience research and clinical applications. Numerous studies have utilized tFUS across various experimental models, including rodent models, non-human primate models, and human subjects (Liu et al., 2021). The technique was first employed for brain modulation in the 1950s, successfully inducing reversible inhibition of sensory evoked potentials in the primary visual cortex of cats via the lateral geniculate nucleus (Fry, 1958). Mihran demonstrated that the mechanical vibrations induced by tFUS could alter cellular excitability in both neuronal and cardiomyocyte populations (Liu et al., 2021). Additionally, Suarez-Castellanos found that tFUS could induce alterations in local field potentials, as assessed through spatiotemporal dynamic microelectrode arrays measuring extracellular neuronal activity in hippocampal regions (Suarez-Castellanos et al., 2021). Yuan et al. (2020) reported that tFUS provoked rapid hemodynamic responses, revealing a linear coupling between cortical cerebral blood flow, local field potentials, and electromyographic amplitude (Yuan et al., 2020). Furthermore, Baek et al. (2018) showed that tFUS increased the amplitude of MEPs and facilitated the gradual restoration of motor function in stroke models, leading to a symmetrical reduction in pathological neural activity and promoting neurological rehabilitation (Baek et al., 2018).

Recent investigations into the molecular effects of tFUS reveal activation of sodium and calcium channels that are critical for neuronal activity (Tyler et al., 2008). Moreover, tFUS has been shown to regulate neurotransmitter levels within the brain, evidenced by significant increases in extracellular dopamine and serotonin concentrations (Liu et al., 2021). In a rat model of Alzheimer’s disease, tFUS increased the expression levels of neurotrophic factors such as BDNF, glial cell-derived neurotrophic factor, and vascular endothelial growth factor through activation of key signaling pathways, which in turn modulated the LTP-like effect and enhanced motor performance (Liu et al., 2017). Notably, tFUS transiently enhances motor cortical excitability when applied to M1, thereby facilitating motor learning successfully integrated EEG (Fomenko et al., 2020; Gibson et al., 2018; Zhang et al., 2021), computed tomography, and fMRI to assess the effects of tFUS in humans, demonstrating its efficacy in modulating deep subcortical regions such as the unilateral thalamus, while achieving impressive spatial accuracy and resolution (Legon et al., 2018).

tFUS has been effectively and safely employed for neuromodulation in small animals, non-human primates, and humans. It is compatible with imaging modalities such as fMRI and computed tomography, showing great potential as a non-invasive neuromodulation technique for treating neurological disorders (Folloni et al., 2019; Legon et al., 2018). Clinical trials have been conducted to evaluate tFUS for conditions including Alzheimer’s disease, Parkinson’s disease, epilepsy, and stroke. Given the absence of non-invasive neuromodulation techniques targeting deep brain structures in clinical settings, tFUS is regarded as a powerful tool within the realm of non-invasive brain stimulation (Fomenko et al., 2020; Meng et al., 2017).

However, despite its advantages in spatial and temporal resolution, achieving high specificity in modulation using ultrasound remains challenging. While numerous studies have validated the safety and efficacy of tFUS, further prospective investigations are required to establish optimal stimulation parameters and delineate safety and effectiveness thresholds (Choi et al., 2020). Future research endeavors should focus on elucidating the cellular, molecular, synaptic, and ionic mechanisms underlying tFUS neuromodulation.

### 3.2 Temporal interference stimulation

**Figure 7:**
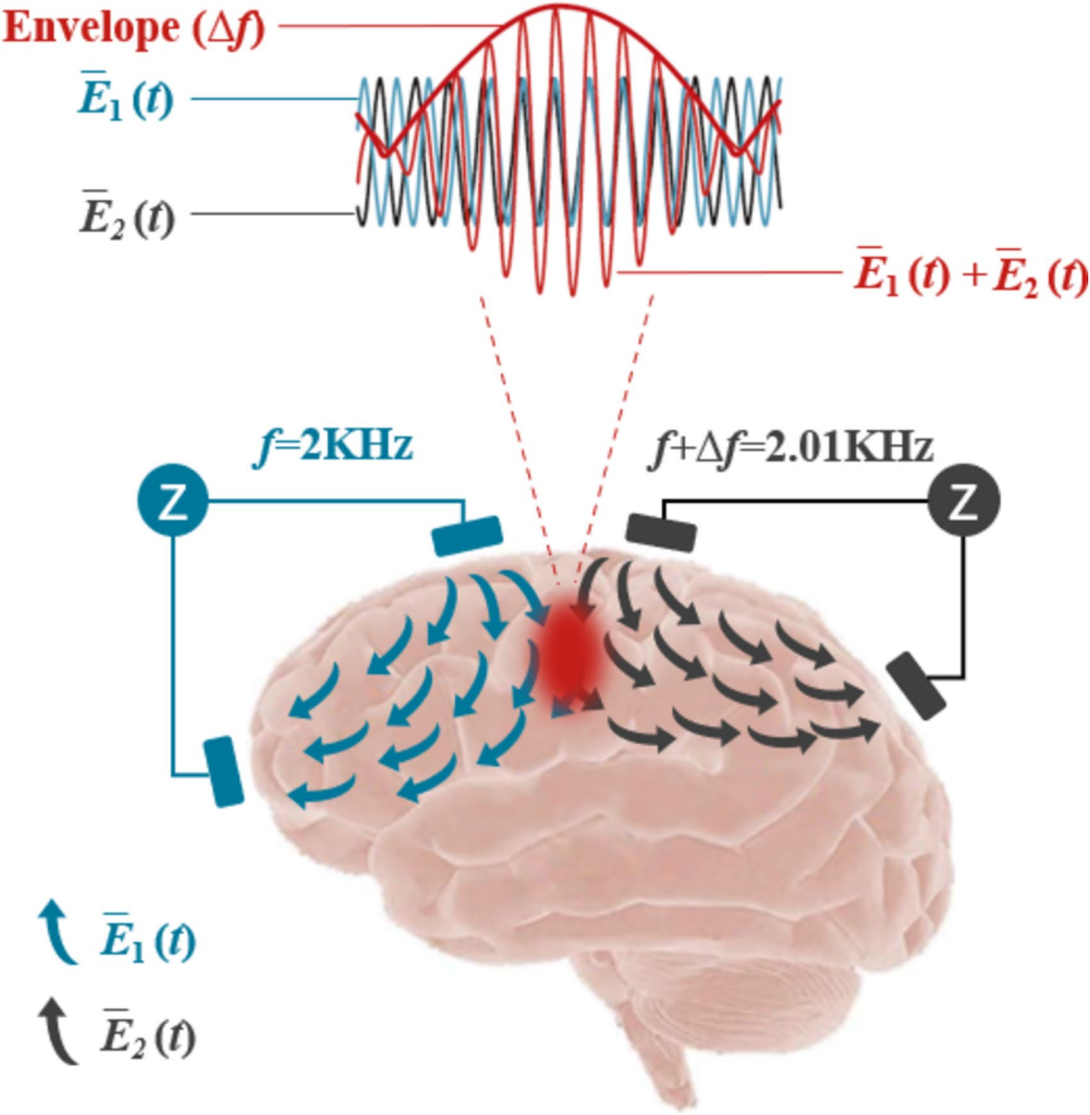
Schematic diagram of the TI principle.

TIS represents a novel non-invasive approach for the targeted modulation of neuronal activity deep within the brain, promising to advance the frontiers of biophysics and neuroscience (Mirzakhalili et al., 2020). As depicted in Figure 7, TIS operated on the principle of interference between two sets of high-frequency AC electric fields, each oscillating at frequencies higher than those typically recorded by EEG. When these two AC fields have a specific frequency difference, they generated a superimposed electric field that produces a low-frequency envelope wave. Importantly, the high-frequency components were insufficient to activate neuronal discharge, due to the long absolute refractory period that separates action potentials, which prevented neurons from responding to high-frequency stimulation (>1,000 Hz). However, the lower frequency envelope wave can effectively drive neuronal activity at a targeted focal point, allowing for the selective stimulation of specific brain regions without impacting adjacent or overlying areas.(Grossman, 2018; Grossman et al., 2017; Grossman et al., 2018).

In experimental applications, Grossman demonstrated the efficacy of TIS by applying high-frequency electric fields at multiple external locations on the mouse brain. When two high-frequency fields with a slight frequency difference (2000 and 2010 Hz) were applied, they produced a low-frequency envelope with a frequency difference (Δ*f*) of 10 Hz, effectively stimulating neurons in deep brain regions such as the hippocampus, while sparing cortical tissues (Dmochowski and Bikson, 2017; Grossman et al., 2017). This precision suggests that TIS offers significant potential for non-invasive, focused brain localization.

Further investigations by Lee utilized a finite element head model of the human brain, targeting the hippocampal region. Their findings indicated that TIS could preferentially direct electric currents to the intended area, optimizing stimulation while simultaneously reducing activity in cortical zones, thereby affirming its capacity for deep neural modulation. This was in line with results from Grossman et al., underscoring the method’s depth and specificity (Lee et al., 2020).

Simulations replicating the conditions of Grossman’s mouse studies further validated TIS’s depth of penetration and efficacy in human anatomical models (Rampersad et al., 2019). Karimi et al. (2019) employed computational analysis using a microscopic model to ascertain the activation region of TIS, demonstrating that the modulation of the electric field could be manipulated through adjustments in electrode positioning and current ratios (Karimi et al., 2019). Their findings corroborated Grossman et al.’s conclusions regarding the potential for deep and adaptable neural stimulation.

Cao and Grover (2020) highlighted how computational modeling could facilitate non-invasive DBS, revealing that TIS exhibits selective efficacy across neurons in mammalian models, warranting caution when translating findings to human applications (Cao and Grover, 2020). In behavioral experiments, Zhang et al. (2021) demonstrated that 70 Hz TIS enhanced motor cortical reaction times and neuronal excitability. Additionally, 20 Hz TIS significantly improved motor learning, showing a positive correlation with increases in motor evoked potentials during serial reaction time tasks (Ma et al., 2021). These studies provide compelling evidence of TIS’s applicability to human motor functions and lay the groundwork for future research.

Recent findings by Zhu et al. (2022) using fMRI indicated that TIS, in conjunction with tDCS, could markedly enhance functional connectivity between the primary motor cortex and secondary motor regions (Zhu et al., 2022). Furthermore, a study showed that TIS applied to right frontoparietal areas in healthy adults could enhance working memory performance (Zhang et al., 2022). Acerbo et al. (2022) confirmed that TIS could be accurately focused on the hippocampus via electric field modeling on human cadavers, with minimal effects on adjacent cortical areas (Acerbo et al., 2022). Additionally, animal studies indicated that TIS could induce physiological changes in an epileptic mouse model, demonstrating its potential as a non-invasive neuromodulation technique for treating neurological disorders.

Our recent studies found that TIS of the left M1 in mice could induce movement in the right forelimb. After a seven-day intervention with an envelope frequency of 20 Hz (Δ*f* = 20 Hz), significant enhancements in motor abilities were observed (Liu et al., 2023). Further work demonstrated that daily TIS (20 min per day for seven consecutive days) at this frequency substantially improved motor skills, potentially through mechanisms involving neurotransmitter metabolism, increased expression of synapse-associated proteins, enhanced neurotransmitter release, and increased dendritic spine density (Qi et al., 2024). This marks the first report detailing the effects of TIS on motor skills in mice and elucidating its underlying mechanisms.

Despite its promise, TIS technology remains in an exploratory phase and faces several challenges. For instance, it does not yet achieve the spatial resolution of implanted DBS techniques (Grossman, 2018; Grossman et al., 2018). Finite element modeling has indicated that TIS can target subcortical structures like the hippocampus or anterior cingulate gyrus but struggles with smaller, deeper brain regions such as the thalamic primordium (Esmaeilpour et al., 2021). While stronger currents can be safely applied at a distance from the scalp, rigorous testing is required to validate such procedures (Grossman et al., 2018). Future advancements may enable tighter focus and deeper penetration by configuring epidural electrodes to bypass current shunts at the scalp-cranial interface (Datta et al., 2009; Grossman, 2018).

In summary, TIS represents a novel non-invasive modality for deep brain stimulation, capable of modulating neuronal activity through strategically applied electric fields across a multi-frequency range. This technique enables targeted stimulation of deep brain structures while preserving cortical integrity, positioning TIS as a valuable tool for functional mapping and therapeutic interventions without the need for electrode repositioning (Grossman, 2018; Grossman et al., 2017). Future research should focus on optimizing the relative amplitudes and positions of electrode pairs to enhance the precision of TIS for targeted therapeutic applications.

## 4 Other novel brain stimulation techniques

TMS methods, such as cortical paired association stimulation (ccPAS), can enhance motor function in young people by increasing the strength of functional connectivity between the ventral premotor cortex (PMv) and the M1 region through spike-time dependent plasticity (STDP) (Turrini et al., 2023). Studies have shown that cc-PAS modulates the dexterity of hand motor functions as well as increases in cortical motor excitability (Rizzo et al., 2011; Turrini et al., 2023). ccPAS can improve homeostasis through a mechanism of facilitated class plasticity in the hyperdirect pathway (Li et al., 2024). ccPAS can also improve metacognitive assessment of organismal sensory responses (Di Luzio et al., 2022). ccPAS as a new technology has shown lasting effects well beyond the duration of NIBS interventions (Hernandez-Pavon et al., 2023). Furthermore, fine-tuning rhythmic TMS (rhTMS) can positively affect cognitive and motor performance (Uehara et al., 2023).

## 5 Challenges

NIBS technologies encounter a variety of challenges that must be addressed to maximize their efficacy and application in both research and clinical settings (see Table 1). Among these technologies, tDCS is one of the most widely utilized due to its non-invasive nature, ease of use, cost-effectiveness, and demonstrated effectiveness. tDCS has found applications in basic neuroscience research, translational clinical studies, and increasingly in military and competitive sports contexts. However, several challenges remain, particularly concerning safety, ethical considerations, and spatial focus.

**Table 1:** Challenges faced by different NIBS.

Type of NIBS	Challenges in sports sciences
tDCS/tACS/tRNS	Safety, ethics and low focus of tDCS applications are the main challenges to face in the future.
TMS	The depth of TMS stimulation is limited and does not allow precise modulation of the deep brain nuclei; the structure of the stimulation coil, the depth and flexibility of stimulation need to be further optimized. In the future, more clinical trials are still needed to provide a basis for scientifically and rationally customizing the parameters of TMS, so as to give full play to the clinical value of TMS.
tFUS	Basic experiments on tFUS should focus on elucidating the potential cellular, molecular, synaptic, and ionic mechanisms of action of tFUS neuromodulation, and further elucidating the stimulus parameters for safety and efficacy of tFUS applications.
TIS	The application of TIS is in the preliminary exploration stage, and the mechanism study still needs more experiments for verification.

Safety concerns are a primary area of focus regarding tDCS. While adverse effects are typically mild—manifesting as transient sensations such as tingling or itching beneath the electrodes—these reactions warrant careful monitoring. Ethical issues surrounding tDCS usage are particularly significant, especially given the absence of clear regulatory guidelines from the World Anti-Doping Agency (WADA) regarding whether tDCS constitutes a “nerve stimulant” that may infringe upon the principles of fair competition in sports. Future research should prioritize establishing safety protocols and defining acceptable usage parameters to protect users, particularly in competitive sports settings. Additionally, the development of precise measuring instruments and methodologies for detecting stimulation effects is essential.

Other NIBS techniques, such as tACS and tRNS, similarly confront challenges related to safety, ethical implications, and limited spatial specificity. Attention must be directed toward refining these techniques to enhance their clinical applicability and effectiveness.

TMS also encounters numerous challenges. One notable limitation is the depth of stimulation; TMS is less effective for directly targeting subcortical structures such as the thalamus and basal ganglia. Further optimization of stimulation coil design, as well as the depth and flexibility of stimulation parameters, is needed to improve efficacy for these deeper brain regions. Additionally, high-frequency TMS can lead to side effects such as headaches or tinnitus. Many studies have reported small sample sizes and inconsistent results, underscoring the need for larger, more robust clinical trials. Such studies are critical for establishing scientifically grounded, customizable TMS protocols that fully leverage its clinical potential.

TI as a relatively new non-invasive brain stimulation technique, faces its own set of challenges. These include technical obstacles inherent in model calculations and animal experiments, which must be navigated before advancing to human applications. Currently, the application of TI in human subjects remains in the preliminary exploratory phase. Further research is necessary to elucidate the underlying mechanisms of TI and to refine its application parameters for optimal effectiveness.

In conclusion, while NIBS technologies like tDCS, TMS, tACS, tRNS, and TIS hold substantial promise for advancing neuroscience and clinical practice (see Table 2), addressing the highlighted challenges is crucial for their successful implementation. Ongoing research efforts must focus on safety, ethical considerations, and optimizing stimulation techniques to enhance the effectiveness and applicability of these innovative methodologies.

**Table 2:** Summary table of key studies.

Classification of brain stimulation techniques	Research theme	Reference
tDCS	tDCS, as a “neural priming technique,” improves athletic performance by improving nerve muscle connections, promoting neuromuscular recruitment, and optimizing coordination between athlete muscle groups.	Hornyak (2017)
tACS	tACS acting on the primary motor cortex of healthy subjects can improve motor learning ability by cross regulating beta oscillatory activity.	Sugata et al. (2018)
rTMS	High frequency rTMS acting on the DLPFC of volleyball players can improve body coordination and enhance athletic performance.	Moscatelli et al. (2023)
rhTMS	As a new method for regulating neural activity rhythm patterns, rhTMS can adjust neural oscillations to target frequencies, which have a positive impact on cognitive and motor performance.	Turrini et al. (2023)
ccPAS	ccPAS can induce functional specific improvement in hand flexibility and increase cortical motor excitability in young people.	Uehara et al. (2023)
tFUS	tFUS modulation of the human brain motor cortex may have long-lasting and statistically significant effects on motor cortex excitability and motor behavior, and no harmful side effects are observed.	Zhang et al. (2021)
TIS	TIS, a novel non-invasive deep brain stimulation, can precisely focus on the deep brain regions of the mouse brain, and different motor patterns can be induced in mice by adjusting the corresponding parameters.	Grossman et al. (2017)

## 6 Conclusion

Various brain stimulation modalities have been shown to have a wide range of applications and potential for improving individual motor performance. Currently, tDCS is most widely used in the field of exercise science, where the technique can enhance muscular strength, explosiveness, and aerobic metabolism, reduce fatigue, and improve cognition, thus becoming a valuable tool for improving athletic performance. However, tDCS is a generalized stimulus that does not precisely modulate specific areas of the brain and deep nuclei responsible for performing motor control and regulation. NIBS represents a promising trajectory for the future of neuromodulation, with emerging technologies like tFUS and temporal interference TIS offering distinct advantages over traditional modalities such as tDCS, tACS, tRNS, and TMS. These advantages include the capability to precisely stimulate deep brain regions while minimizing interference with cortical function, thereby achieving higher spatial and temporal resolution in neuromodulation. The advancement of these novel techniques is expected to overcome limitations associated with conventional approaches and facilitate accurate modulation of deep brain structures. Future research should prioritize multidisciplinary collaborations across fields such as clinical medicine, biomedical engineering, neuroscience, computer science, and sports science to drive the development of NIBS technologies.

## Data Availability

The original contributions presented in the study are included in the article/supplementary material, further inquiries can be directed to the corresponding authors.
